# Enhancing Job Satisfaction for Oncology Staff: Research Versus Inquisitive Conversations

**DOI:** 10.1007/s13187-024-02495-w

**Published:** 2024-08-28

**Authors:** Rajiv Samant

**Affiliations:** Division of Radiation Oncology, The Ottawa Hospital Cancer Centre, Ottawa, ON Canada

**Keywords:** Job satisfaction, Wellness

## Abstract

The burden of cancer is increasing, and this is putting incredible strain on the healthcare system, specifically on frontline oncology health care providers. This is well documented in the published literature, and the situation has been exacerbated following the COVID-19 pandemic. There is ample evidence about the concerns of healthcare staff as to what changes are needed to improve their work lives and make them more efficient and effective at their jobs. There can always be more research done in this area, but, more importantly, there is an urgent need to start taking concrete steps and appropriate action to improve the situation so cancer patients can get the best available care possible.

The burden of cancer is increasing worldwide, and this requires that healthcare systems be ready and prepared. Well-trained professionals in oncology are essential for providing the necessary care, and suitable conditions have to be in place for them to work efficiently and effectively [1]. Therefore, good quality care for cancer patients hinges on oncology staff being appropriately trained and supported [2]. Over the past decade, there have been numerous reports about the high rates of stress, dissatisfaction, and burnout among healthcare providers, including those working in oncology; and the situation has only become worse after the COVID-19 pandemic [3]. Resources have been invested to research the problems being encountered, and various approaches to manage the situation have been postulated, especially from an academic perspective [4]. Our Cancer Center has also participated in such a process and published our results [5]. However, when do we start to focus more on dealing with the problems at hand, and which are quite consistent in the literature? Although research is important, the focus should be on listening to what is being said and making a sincere and concerted effort to make improvements. In the summer of 2019, I was part of an ad hoc quality improvement (QI) group established to look at ways to assess and improve staff well-being and job satisfaction within the oncology and hematology realm. As part of this initial process, I interviewed five staff members at our Cancer Center, with representation from nursing, radiation therapy, and physicians. These interviews basically consisted of semi-structured conversations, specifically asking about various aspects of job satisfaction: likes, dislikes, and suggestions for improvement. Unfortunately, like many promising projects, this initiative fizzled as the COVID-19 pandemic took hold. Since then, there have been managerial changes within our organization, and this initiative has been long forgotten. Fast forward to late 2023, we published our Cancer Center staff satisfaction survey research project, which took hundreds of hours and several years to prepare, administer, analyze, and publish. We have been proud of our efforts and over 450 respondents participated in the study. In the end, we summarized the key messages from our research, and it was consistent with most of our expectations and similar to what has been published in the medical literature [5]. We submitted our findings to management and have been awaiting the next steps, because all of the work is really moot if it does not lead to meaningful changes and does not help improve the workplace environment for staff. In 2024, as I went through my old e-mail and Word documents, I came across the interview summaries from 2019. I reviewed the core messages from the small sample size of staff and was surprised at how remarkably similar they were to our main research findings as shown in Table 1.

**Table 1:** Comparison of interview and research survey responses

Themes	Interviews	Survey
Promote job satisfaction	-Teamwork/collaboration/supportive colleagues -Patient contact and connection and the ability to help others -Acknowledgement/recognition for good work -Manageable schedules and workload -Having adequate resources	-Working with colleagues -Working with patients -Learning new things -Personal achievements -Acknowledgement from others -Supportive work environment -Manageable workload -Financial compensation -Academics
Challenges at work	-Stress/burnout/heavy workload/not enough protected time -Inadequate individual recognition or acknowledgement for the work being done -Inadequate/inefficient clinic processes (sometimes quantity appears to be more important than quality) -Too many bureaucratic tasks -Unsupportive managers/supervisors	-Technology problems -Unsupported work environment -Heavy workload -No future prospects -Inadequate clinic design -Too much paperwork -Inadequate financial compensation -Verbal/physical harassment
Suggestions for improvement	-More team-focused approach -Appropriate working conditions and resources (including staffing, space, time, scheduling) -Workplace autonomy and flexibility -Supportive leaders and managers -More individual recognition and positive feedback -Protected time for non-clinical work -More efficient workflow -Allow for staff development	-Workplace flexibility -Manageable workload -More supportive management -More acknowledgement and respect -Fewer meetings–better staff engagement -Better staff rooms -More teamwork -Better communication -Increased remuneration -More education and training

Does that mean that all our research efforts were a waste of time? Not necessarily. However, I think it does show that inquisitive conversations with representative staff done in an anonymous, non-judgmental, and empathetic manner can, at times, be just as powerful as formal and systematic research. Every cancer center or healthcare facility does not have to do novel and original research, or pore through the latest publications, to figure out how to improve the lives of their staff. They just need to allow for meaningful dialogue and sincerely listen to what is being said by staff. Most importantly, they need to take stock of what is being said and put in the effort to find workable solutions. And thus, improving job satisfaction among oncology staff should lead to better care for our cancer patients.
